# The Additive Value of Femoral Ultrasound for Subclinical Atherosclerosis Assessment in a Single Center Cohort of 962 Adults, Including High Risk Patients with Rheumatoid Arthritis, Human Immunodeficiency Virus Infection and Type 2 Diabetes Mellitus

**DOI:** 10.1371/journal.pone.0132307

**Published:** 2015-07-31

**Authors:** Athanasios D. Protogerou, Jaap Fransen, Evangelia Zampeli, Antonis A. Argyris, Evagelia Aissopou, Aikaterini Arida, George D. Konstantonis, Nikos Tentolouris, Konstantinos Makrilakis, Mina Psichogiou, George Daikos, George D. Kitas, Petros P. Sfikakis

**Affiliations:** 1 First Department of Propaedeutic & Internal Medicine, Laikon Hospital, Athens, Greece; 2 Joint Academic Rheumatology Program of the National and Kapodistrian University of Athens Medical School, Athens, Greece; 3 Department of Rheumatology, Radboud University Medical Center, Nijmegen, The Netherlands; 4 Department of Rheumatology, Dudley Group NHS Foundation Trust, Dudley, United Kingdom; 5 Arthritis Research UK Epidemiology Unit, University of Manchester, Manchester, United Kingdom; University of Perugia, ITALY

## Abstract

**Background:**

Presence of femoral atheromatic plaques, an emerging cardiovascular disease (CVD) biomarker additional to carotid plaques, is poorly investigated in conditions associating with accelerated atherosclerosis such as Rheumatoid Arthritis (RA), Human Immunodeficiency Virus (HIV) infection and Type 2 Diabetes Mellitus (T2DM).

**Objective/Methods:**

To assess the frequency of femoral/carotid subclinical atheromatosis phenotypes in RA, HIV and T2DM and search for each disease-specific probability of either femoral and/or carotid subclinical atheromatosis, we examined by ultrasound a single-center cohort of CVD-free individuals comprised of consecutive non-diabetic patients with RA (n=226) and HIV (n=133), T2DM patients (n=109) and non-diabetic individuals with suspected/known hypertension (n=494) who served as reference group.

**Results:**

Subclinical atheromatosis - defined as local plaque presence in at least on arterial bed - was diagnosed in 50% of the overall population. Among them, femoral plaques only were found in 25% of either RA or HIV patients, as well as in 16% of T2DM patients and 35% of reference subjects. After adjusting for all classical CVD risk factors, RA and HIV patients had comparable probability to reference group of having femoral plaques, but higher probability (1.75; 1.17 - 2.63 (odds ratio; 95% confidence intervals), 2.04; 1.14 - 3.64, respectively) of having carotid plaques, whereas T2DM patients had higher probability to have femoral and carotid plaques, albeit, due to their pronounced dyslipidemic profile.

**Conclusion:**

RA and HIV accelerate predominantly carotid than femoral. A “two windows” carotid/femoral, rather than carotid alone ultrasound, screening improves substantially subclinical atheromatosis detection in patients at high CVD risk.

## Introduction

Accelerated carotid atheromatosis has been widely described in the presence of classical cardiovascular disease (CVD) risk factors, as well as in patients with Rheumatoid Arthritis (RA) [1,2], Human Immunodeficiency Virus (HIV) infection [3] and Type 2 Diabetes Mellitus (T2DM) [4]. In these populations, atheromatosis should be regarded as an important pathophysiological mechanism associated to their elevated CVD risk [4,5,6,7]. Subclinical carotid atheromatosis is a predictor of mortality, independently from major classical CVD risk factors, in individuals with hypertension or diabetes [8], those with established CVD [9], as well as in patients with specific diseases such as RA [10] and HIV infection [11]. Thus, carotid plaque presence might serve as a vascular biomarker to optimize CVD risk prediction. Indeed, several recent international guidelines have incorporated carotid ultrasound examination for the optimized reclassification of CVD risk [4,12,13,14,15,16].

Atheromatosis is a pathological process affecting multiple arterial sites, yet in an unpredictable pattern. Most recently, Weir-McCall et al. showed that subclinical carotid atheromatosis does not correlate with global atheromatosis burden [17]. In clinical practice, assessment of subclinical femoral atheromatosis is easy but much less investigated than carotid atheromatosis. Recent data have shown that the combined presence of both carotid and femoral plaques associates more strongly than carotid plaque alone with the incidence of CVD events in low CVD risk individuals [18], in patients with Systemic Sclerosis and Systemic Lupus Erythematosus [19]. Moreover, several lines of evidence suggest that classical CVD risk factors affect differentially atheromatosis in distinct arterial sites (e.g. carotid and femoral arteries)[20]. Therefore, it is important to know whether, by using a “two-windows” (femoral and carotid) subclinical atheromatosis screening approach, we can detect a substantial number of patients in each disease that will have either the unfavorable phenotype of combined femoral and carotid subclinical atheromatosis, or, will have only femoral subclinical atheromatosis that would not be identified by the one window (carotid) screening approach. Although carotid subclinical atheromatosis has been investigated in large cohorts [21], on the contrary, studies investigating both subclinical femoral and carotid atheromatosis especially in the presence of novel CVD risk factors, such as RA disease [22] and HIV infection [23] arescarce.

Based on the above, in the present study we aimed: (a) to assess the frequency of femoral/carotid subclinical atheromatosis phenotypes (i.e. only femoral; only carotid; both femoral and carotid) in patients with RA, HIV infection and T2DM; and (b) to assess the disease-specific probability of either carotid and/or femoral plaque presence, compared to a reference group of non-diabetic individuals with suspected/known hypertension.

## Methods

All participants provided written informed consent according to the World Health Organization statement on ethical principles for medical research involving human subjects developed in Helsinki [24] and the protocol was approved by the “Laiko” Hospital’s institutional review board.

### Study design—Population

Consecutive consenting adult patients with:(i) RA (without T2DM) from the Rheumatology outpatient clinic; (ii) HIV infection (without T2DM) from the HIV outpatient clinic;(iii) T2DM (without any chronic inflammatory or autoimmune disease) from the Diabetes outpatient clinic, were all referred to the Cardiovascular Research Laboratory of our department and included in the study. Moreover, individuals with suspected or diagnosed hypertension (without diabetes and any chronic inflammatory/autoimmune disease)from the Hypertension outpatient clinic were included in the study and comprised the RG. All participants underwent the same vascular investigations. Participants with established CVD (defined as pre-existing coronary artery disease, stroke and peripheral arterial disease), type 1 diabetes mellitus and any other than the predefined chronic diseases were excluded from the analysis, in order to avoid any confounding effect. All RA patients had a clinical diagnosis and met the retrospective application of the American Rheumatism Association 1987 revised criteria [25]. HIV was diagnosed on the basis of the ECDC Guidance HIV testing guidelines [26]. T2DM was diagnosed by use of glucose lowering drugs or abnormal fasting glucose levels >125mg/dl in 2 separate occasions and/or HbA1c>6.5% and/or abnormal oral glucose tolerance test [27].

### Arterial ultrasound studies

In this CVD disease-free population, subclinical atherosclerosis was defined as the presence plaque in either the femoral or carotid bed. High-resolution ultrasound was used for the detection of carotid artery and femoral artery plaques in 8 (far and near wall) arterial sites (left and right common carotid, internal carotid arteries and carotid bulb and common femoral artery). Atheromatic plaques were defined as local increase of the intimal-medial thickness of >50% compared to the surrounding vessel wall, an intimal-medial thickness>1.5 mm, or local thickening >0.5 mm [28]. Carotid plaque presence was defined as the presence of plaque in at least 1 of the 6 measured carotid sites. All measurements were performed by the same experienced operator (GD Konstantonis) using a high-resolution B-mode ultrasound device (Vivid 7 Pro, GE Healthcare) with a 14-MHz multi-frequency linear transducer.

### Definition of CVD risk factors

Hypertension was defined by the use of antihypertensive drugs and/or office blood pressure measurement higher than 139/89 (average of 3 sequential readings with 1 min interval in the supine position after at least 10 min of rest (Microlife WatchBP Office, Microlife AG, Widnau, Switzerland). Dyslipidemia was defined on the basis of treatment with lipid modifying drugs or low-density lipoprotein cholesterol level >160 mg/dl. Current smoking was defined by the use of at least 1 cigarette per day each day of the week; ex smoking was defined as discontinuation for more than 6 months. Body mass index was calculated as weight/(height^2^) (kg/m^2^) and used as a marker of obesity. Family history of premature CVD was defined as the presence of coronary heart disease in a 1st degree relative under the age of 55 years for males and 65 years for females. Required data were retrieved from the medical records of the participants.

### Statistical analysis

Continuous variables are presented as mean±standard deviation and categorical variables as percentage. We used multivariate logistic regression models (with odds ratio and 95% confidence intervals), before and after correcting for the presence of intermediates/confounders, to test the probability in each of the above subgroups of patients (T2DM, RA and HIV infection) to have either carotid or femoral subclinical atheromatosis (plaque presence) using as comparator the RG. The selection of CVD risk factors as potential intermediates/confounders was based on their clinical use as suggested by 7 worldwide recommendations for screening for total CVD risk [29]. The major classical CVD risk factors (i.e. those recommended by all 7 guidelines [29] i.e. age, gender, smoking, dyslipidemia, hypertension were used in the models, as follows: model 1: unadjusted; model 2: adjusted for age and sex; model 3: model 2& smoking; model 4: model 3 & dyslipidemia; model 5: model 4 & hypertension. Additionally the next two most widely applied CVD risk factors i.e. body mass index for obesity and family history for premature coronary heart disease were introduced in model 6. Finally, in model 7 further adjustments were performed for the presence of blood pressure-lowering drugs, lipid-modifying drugs and glucose-lowering drugs. P< 0.05 was considered as the level of statistical significance. SPSS version 22.0 was used for all statistical analyses.

## Results

A total of962participants were included in the analysis (Table 1). Of them 226 had RA disease (time from diagnosis123.7 (103.3–139.9) months), 133 had HIV infection (time from diagnosis 63.1 (52.2–73.9) months), 109 had T2DM, and 494 comprised the reference group. RA patients were predominantly women, older, less often dyslipidemic and were less often smokers compared to the RG. HIV infected patients were predominantly men, younger, less obese and more often smokers, but less often hypertensives and dyslipidemic, than the RG individuals. Diabetics were older, more obese and had more often hypertension and dyslipidemia than the RG.

**Table 1:** Descriptives of the population. Continuous variables are presented as mean±standard deviation and categorical variables as percentage.

	RG(n = 494)	T2DM(n = 109)	HIV(n = 133)	RA(n = 226)
Age (years)	52.2±13.5	61.5±8.4	40.5±11.5	57.7±12.2
Male gender (%)	50.8	49.5	89.5	18.1
Hypertension (%)	59.7	78.9	15.8	51.8
Dyslipidemia (%)	37.7	66.1	26.3	28.8
Smoking				
Current (%)	33.6	38.5	52.6	29.2
Ex (%)	20.6	25.7	14.3	21.2
Family coronary heart disease (%)	14.8	16.7	15.9	14.3
Body mass index (kg/m^2^)	27.5±4.9	31.1±5.3	24.5±3.6	26.8±5.2
Drugs for diabetes (%)	0	94.5	0	0
Drugs for hypertension (%)	38.5	67.9	6.0	38.1
Drugs for dyslipidemia (%)	18.0	55.0	10.5	14.6

Abbreviations: RG: reference group, T2DM: type 2 diabetes mellitus, HIV: human immunodeficiency virus, RA: Rheumatoid arthritis.

### Presence of carotid and/or femoral subclinical atheromatosis

**Fig 1:**
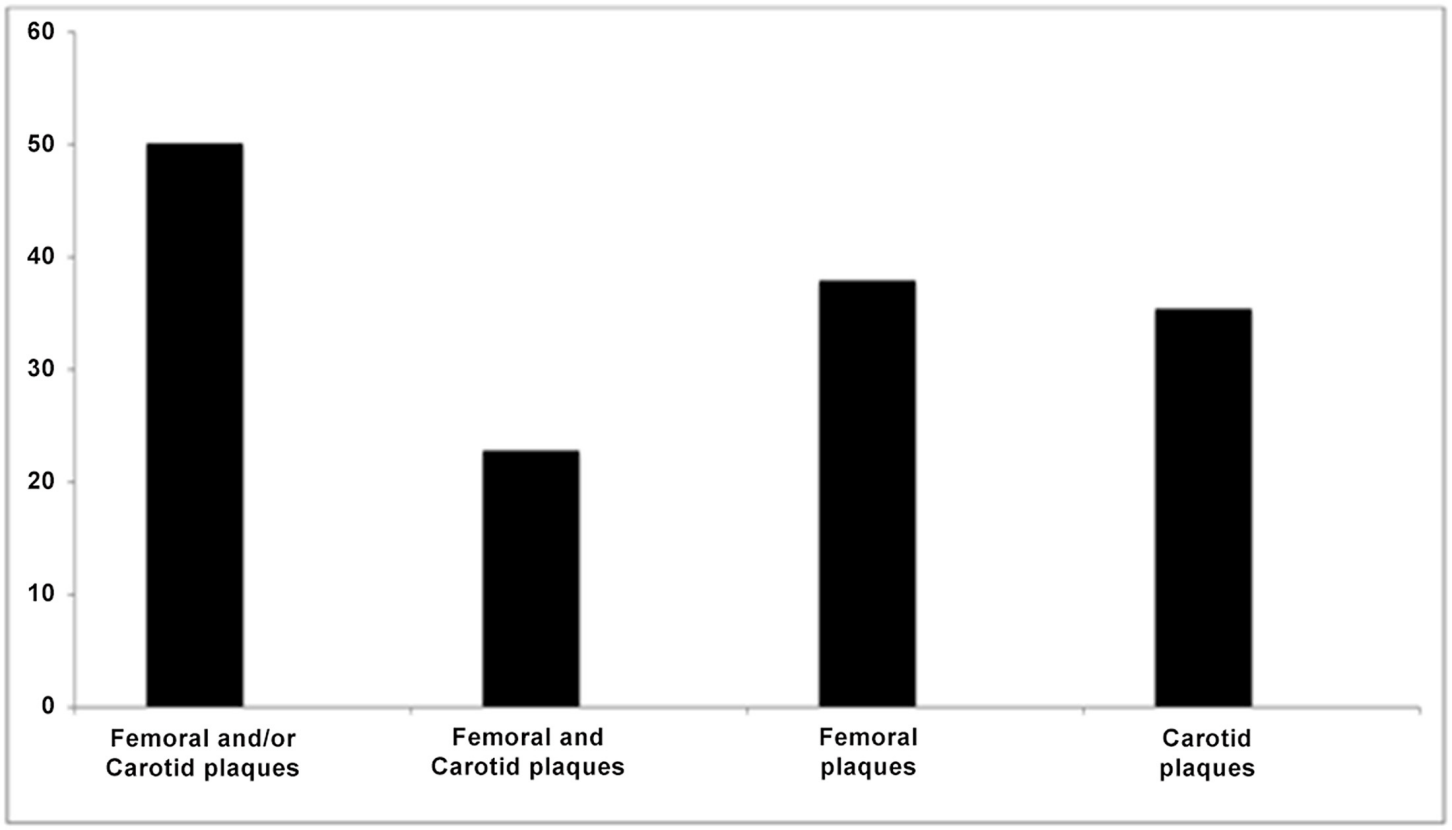
Prevalence of subclinical atheromatosis assessed by plaques in femoral and carotid arteries in the studied adult cohort of 962 patients.

In the total cohort, 37.9% had at least one plaque at the femoral bed; 35.4% had at least one plaque at the carotid artery; 22.8% had plaques both at the femoral and the carotid beds; 50.1% had at least one plaque either at the carotid or the femoral artery or in both (Fig 1).

**Fig 2:**
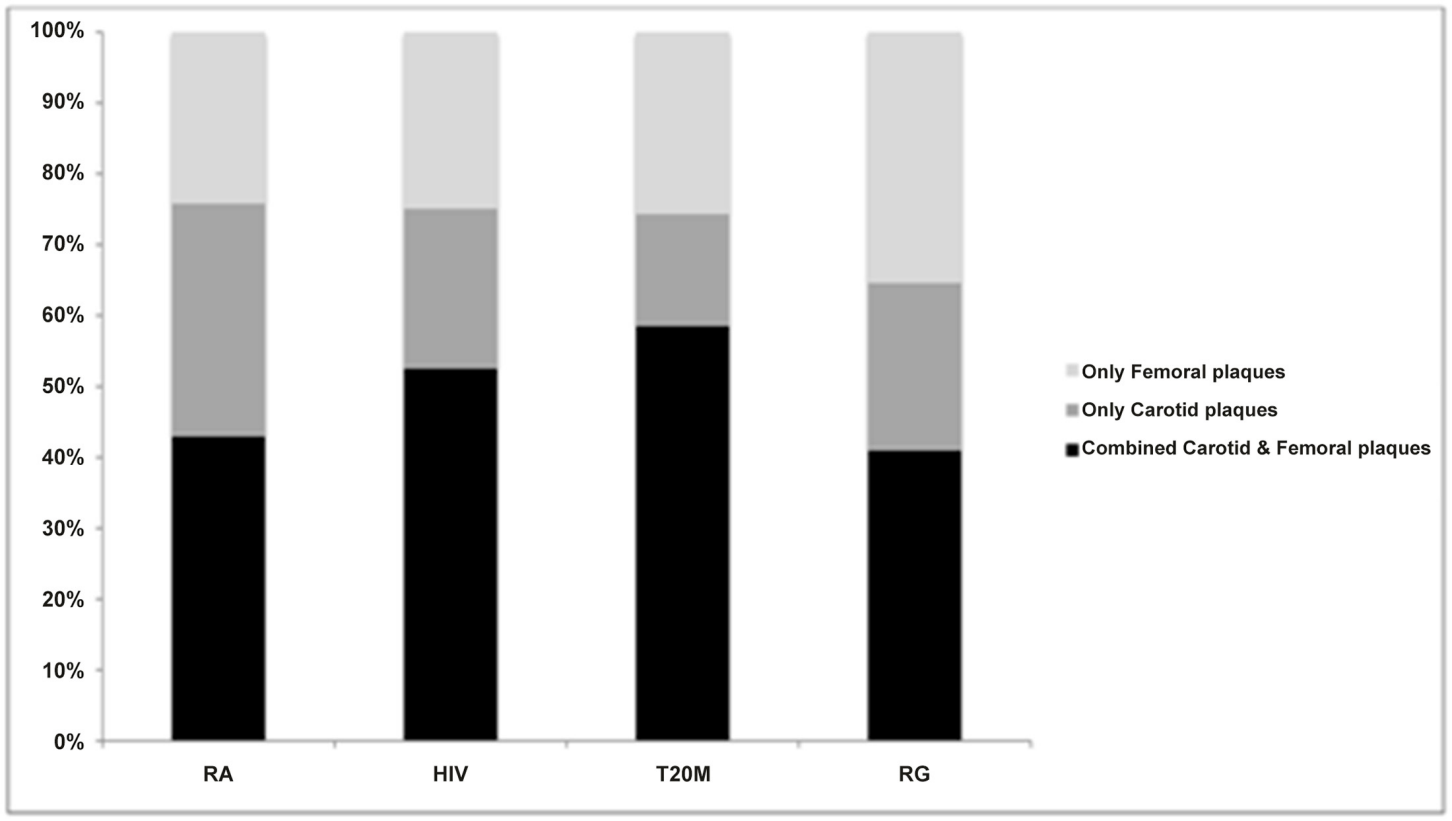
Distribution (%) of plaques (i) only in carotid, (ii) only in femoral or (iii) in both carotid and femoral arteries in individuals with subclinical atheromatosis associated with rheumatoid arthritis (RA), human immunodeficiency virus (HIV), and type 2 diabetes mellitus (T2DM) and in the reference group(RG). (Abbreviations: DM: diabetes mellitus, HIV: human immunodeficiency virus, RA: Rheumatoid arthritis.)

Among those participants with subclinical atheromatosis defined by the presence of any plaque in the carotid or the femoral bed (50.1% of the total cohort) (i) only femoral plaques, (ii) only carotid plaques, (iii) both femoral/carotid plaques were observed in: 29.9%, 24.7%, 45.4% in the total cohort; 24.2%, 32.9%, 42.9% in the RA subgroup; 24.9%, 22.6%, 52.5% in the HIV subgroup; 25.7%, 15.8%, 58.5% in T2DM and 35.4%, 23.7%, 40.9% in the RG (Fig 2).

### Probability of patients with T2DM, RA and HIV to have carotid plaques and femoral plaques compared to RG

After adjusting step by step (models 1 to 7, Table 2) for non-modifiable (age, sex, family history of premature coronary heart disease) and modifiable (smoking, hypertension, dyslipidemia, obesity) CVD risk factors, patients with RA and HIV infection had higher probability by 1.75 (1.17–2.63) and 2.04 (1.14–3.64) (odds ratio; 95% confidence interval), respectively, to have carotid plaques compared to RG (model 6, Table 2). In contrast, patients with RA and HIV infection did not have statistically significantly higher probabilities than RG to have femoral plaques (1.33 (0.87–2.02) and 0.70 (0.40–1.22), respectively (Table 2). Patients with T2DM had higher probability by 1.67 (1.05–2.67) and 1.91 (1.16–3.16) than RG to have carotid plaques and femoral plaques, respectively (model 3); however after adjustment for the presence of dyslipidemia this association lost statistical significance (Table 2).

**Table 2:** Probability of patients with Type 2 Diabetes Mellitus, Rheumatoid Arthritis and Human Immunodeficiency Virus Infection to have carotid plaque and femoral plaque (odds ratio (95% confidence interval)) in multivariate regression analysis using as comparator the reference group, before and after adjustment for potential confounders (model 1 to model 7).

		Carotid plaque(n = 962)	Femoral plaque (n = 958)
**Model 1 (unadjusted)**	Type 2 Diabetes Mellitus	**3.14 (2.05–4.81)**	**3.25 (2.10–5.03)**
	Human immunodeficiency virus infection	0.67 (0.43–1.05)	0.54 (0.35–0.85)
	Rheumatoid Arthritis	**1.76 (1.27–2.42)**	1.12 (0.81–1.54)
**Model 2 (age & sex)**	Type 2 Diabetes Mellitus	**1.84 (1.16–2.91)**	**2.14 (1.32–3.46)**
	Human immunodeficiency virus infection	1.52 (0.90–2.58)	0.74 (0.44–1.22)
	Rheumatoid Arthritis	**1.48 (1.02–2.16)**	**2.13 (1.32–3.46)**
**Model 3 (model 2 & smoking)**	Type 2 Diabetes Mellitus	**1.67 (1.05–2.67)**	**1.91 (1.16–3.16) 5.03-**
	Human immunodeficiency virus infection	1.52 (0.89–2.62)	0.70 (0.41–1.18)
	Rheumatoid Arthritis	1.44 (0.98–2.12)	1.16 (0.78–1.73)
**Model 4 (model 3 & dyslipidemia)**	Type 2 Diabetes Mellitus	1.58 (0.98–2.53)	1.62 (0.97–2.69)
	Human immunodeficiency virus infection	1.51 (0.88–2.60)	0.69 (0.40–1.18)
	Rheumatoid Arthritis	**1.51 (1.02–2.22)**	1.34 (0.89–2.01)
**Model 5 (model 4 & hypertension)**	Type 2 Diabetes Mellitus	1.52 (0.94–2.44)	1.59 (0.96–2.64)
	Human immunodeficiency virus infection	**2.00 (1.14–3.51)**	0.75 (0.43–1.31)
	Rheumatoid Arthritis	**1.68 (1.12–2.50)**	1.39 (0.92–2.10)
**Model 6** * **(model 5 & BMI, fCHD)**	Type 2 Diabetes Mellitus	1.56 (0.95–2.55)	1.58 (0.93–2.67)
	Human immunodeficiency virus infection	**2.04 (1.14–3.64)**	0.70 (0.40–1.22)
	Rheumatoid Arthritis	**1.75 (1.17–2.63)**	1.33 (0.87–2.02)

* Further adjustment (model 7) for blood pressure lowering drugs, lipid modifying drugs and glucose lowering drugs did not modify the results.

BMI: body mass index, fCHD: family history of premature coronary heart disease

### The effect of classical CVD risk factors on carotid and femoral plaques

In multivariate regression analysis (model 6, Table 3), older age, male sex and smoking (current or ex-) were associated with higher probability for the presence of both carotid and femoral plaques; hypertension only with higher probability for carotid plaque presence and dyslipidemia only with higher probability for femoral plaque presence (Table 3).

**Table 3:** Associations of classical and novel cardiovascular disease risk factors with the presence of carotid plaque and femoral plaque (odds ratio (95% confidence interval)) in multivariate regression analysis (model 6 of table 2).

	Carotid plaque	Femoral plaque
Type 2 Diabetes Mellitus	1.56 (0.95–2.55)	1.58 (0.93–2.67)
Human immunodeficiency virus infection	**2.04 (1.14–3.64)**	0.70 (0.40–1.22)
Rheumatoid Arthritis	**1.75 (1.17–2.63)**	1.33 (0.87–2.02)
Confounders in the adjusted model *		
Age (for 1 year increase)	**1.09 (1.07–1.12)**	**4.84 (3.24–7.18)**
Male gender	**1.49 (1.03–2.14)**	**2.50 (1.62–3.85)**
Smoking		
Current (vs. non-smokers)	**3.02 (2.05–4.43)**	**4.84 (3.24–7.18)**
Ex (vs. non-smokers)	**1.78 (1.16–2.72)**	**2.50 (1.62–3.85)**
Hypertension	**2.10 (1.46–3.02)**	1.30 (0.90–1.88)
Dyslipidemia	1.29 (0.93–1.78)	**2.48 (1.78–3.56)**
Family history of coronary heart disease	1.41 (0.92–2.17)	1.05 (0.67–1.63)
Body mass index (for 1 Kg/m^2^ increase)	1.00 (0.97–1.04)	0.99 (0.95–1.02)

* Further adjustment for blood pressure lowering drugs, lipid modifying drugs and glucose lowering drugs did not modify the results.

In order to better adjust for age and gender we performed subanalysis using the final fully adjusted regression model (i.e. model 6) separate for each disease (i.e. for T2DM by excluding HIV subgroup and RA group; for HIV by excluding T2DM and RA; for RA by excluding T2DM and HIV). We excluded males in case of RA analysis and we excluded females in case of HIV analysis. In each subanalysis frequency matching for age between the reference group and each test group was performed. The results in each subanalysis regarding each test group (disease group) shoed overall similar results to the main analysis (data not shown).

### Probability of patients with T2DM, RA and HIV to have “both carotid and femoral plaques” or “either carotid or femoral plaque” compared to RG

In order to test the probability of each disease to have a composite of “both carotid and femoral plaques” or “either carotid or femoral plaque we repeated the regression analysis testing the odds ratio of having a composite of “both carotid and femoral plaques” versus “no plaque at all”, as well as, of having “either carotid or femoral Plaque” vs. “no plaque at all”. This analysis (Table 4) verified the differences between the main diseases (T2DM, HIV and RA) regarding their association with subclinical atheromatosis phenotypes and particularly showed that: (i) RA disease is associated with higher probability to develop “combined carotid & femoral plaques” as well as isolated atheromatosis (either at the carotid or the femoral bed); (ii) T2DM is associated with higher probability to develop “combined carotid & femoral plaques” but not isolated atheromatosis (either at the carotid or the femoral bed); (iii) most classical CV risk factors increased the odds ratio of having “isolated carotid or femoral plaque” as well as of “combined carotid and femoral plaques”.

**Table 4:** Associations of classical and novel cardiovascular disease risk factors with the presence of either “carotid plaque or femoral plaque” and “both carotid and femoral plaque” (odds ratio (95% confidence interval)) in multivariate regression analysis (model 6 of table 2).

	Carotid or femoral plaque	Carotid & femoral plaques
Type 2 Diabetes Mellitus	1.18 (0.65–2.29)	**2.49 (1.25–4.96)**
Human immunodeficiency virus infection	0.69 (0.36–1.32)	1.60 (0.75–3.43)
Rheumatoid Arthritis	**1.95 (1.23–3.10)**	**1.90 (1.09–3.50)**
Confounders in the adjusted model		
Age (for 1 year increase)	**+**	**+**
Male gender	**+**	**+**
Smoking		
Current (vs. non-smokers)	**+**	+
Ex (vs. non-smokers)	**+**	**+**
Hypertension	+	+
Dyslipidemia	**+**	**+**
Family history of coronary heart disease	**-**	**-**
Body mass index (for 1 Kg/m^2^ increase)	**-**	**-**

((+): in the model, (-): not in the model).

## Discussion

Three are the novel findings of this study. First, variable but significant proportions of patients with high CVD risk-associated conditions such as RA, HIV infection and T2DM have subclinical atheromatosis only in the femoral arteries, irrespective of the underlying condition and the CVD risk factors. Variability is partly due to the accumulation of different classical CVD risk factors in each disease that—as the present data showed—differently affect femoral or carotid atheromatosis. For example, the vast majority of HIV patients, who are predominantly men and younger that RA and T2DM patients, consists of smokers. Second, chronic inflammatory conditions such as RA and HIV infection accelerate mainly carotid, rather than femoral subclinical atheromatosis. Third, a “two windows” (i.e. both femoral and carotid) screening approach for the detection of asymptomatic arterial plaque presence, would detect: (i) 25%of individuals with isolated femoral atheromatosis, and (ii) almost half of those who are at higher CVD risk due to presence of combined femoral and carotid plaques.

The main strengths of the present study lie to the fact that it is a single-center study including almost 1000 participants with or without classical and novel CVD risk factors such as RA and HIV infection, in whom subclinical atheromatosis at both the femoral and carotid bed was simultaneously assessed by the same operator.

In accordance with previous studies [1,2,3,30,31,32] we found that RA disease and HIV infection are associated with 1.8 and 2.0 times higher probability of subclinical atheromatosis in the carotid bed, respectively. Data from patients undergoing endarterectomy have shown that carotid plaques had significantly increased proportions of macrophages/T cell infiltrates compared to femoral plaques, suggesting a more inflammatory phenotype in the carotid arterial bed [33]. Furthermore, carotid plaques predominantly displayed M1-activation markers, whereas femoral plaques M2-macrophages, the latter being associated with better plaque stability traits than the former [33]. These data imply that inflammation may play a more important role in the atheromatous process and plaque vulnerability in the carotid than the femoral bed. Considering the chronic inflammatory nature of both RA and HIV diseases, this hypothesis might explain our findings and should be further examined in RA and HIV patients, as well in other chronic autoimmune-mediated inflammatory diseases.

Several lines of evidence have previously shown that there are major differences regarding the atherogenic effect of classical CVD risk factors between the femoral and carotid bed [20,34,35,36]. We found no difference regarding the effect of age on the two arterial beds but men had higher relative probabilities of having femoral than carotid plaques, whereas inversely women had higher relative probabilities of having carotid than femoral plaques. Hypertension was also associated with higher relative probabilities to have carotid than femoral plaques whereas current smoking and dyslipidemia were associated with higher relative probabilities to have femoral than carotid plaques. Of note, in the present study the equal effect of T2DM on both arterial beds was predominantly mediated by the presence of dyslipidemia and not by glycaemia per se.

Previous studies have shown that differences between the two arterial beds exist also regarding the type of arterial remodelling process [37,38] as well as the morphological and histological traits of the plaques [33,39,40]. Even more the response to statin treatment differs between the two beds and is modulated by the presence of other CVD risk factors [41,42]. The clinical implications of all these differences have not been scrutinized yet but seem important. Subclinical femoral atheromatosis is independently associated with CVD incidence [43] and associates with the presence of CVD disease better than carotid atheromatosis [44,45,46,47]. This observation is related to emerging data suggesting that, compared to the other arteries (brachial, common carotid and ascending aorta) the femoral artery has an atherogenicity profile [48] and arterial wall elastic properties [49,50] that are closer to those of the coronary arterial bed. Therefore, it might be used as a better proxy of the coronary bed than the carotid artery [51]. Most importantly, by detecting patients with combined carotid and femoral subclinical atheromatosis it is feasible to identify the population at the highest CVD risk [18,19,46,52].

Because the existing CVD prediction scores may underestimate CVD risk in specific populations such as RA and HIV, international societies have proposed the incorporation of these novel CVD risk factors in the existing CVD risk prediction models in order to improve their discriminatory ability [53,54,55,56,57]. The performance of this strategy is under investigation. An alternative or complementary way to go forward is the addition of vascular biomarkers, such as subclinical plaque presence, in these models [4,12,13,14,15,16]. This approach may have additional advantages since subclinical atheromatosis integrates information related to all (novel and classical) CVD risk factors, and other yet poorly identified environmental and genetic factors [58]. In view of the above, physicians involved in primary CVD prevention of patients with novel CVD risk factors, such as RA disease or HIV infection, should be aware of the fact that, although these specific chronic inflammatory immune-mediated diseases may affect predominantly carotid atheromatosis, the accumulation of different comorbidities (such as smoking, dyslipidemia or hypertension) lead to acceleration of atheromatosis in other arterial beds.

The present study should be interpreted keeping in mind the following limitations. We acknowledge the fact that study groups differ in other aspects (age, gender) than disease (RA, DM, HIV) alone. Therefore, we paid considerable attention to statistical correction for confounders, as described in the methods and as shown in Tables 2 and 3. Statistical correction using multivariate models is the method of choice in observational research, matching (e.g. for age and gender) is no advantage above statistical correction. The present analysis did not aim to investigate the specific mechanisms that lead to acceleration of atheromatosis in each one of the main studied pathologies (RA, HIV infection and T2DM) and did not investigate differences between them regarding plaque characteristics and stability. To what extend atheromatosis is caused by the dissimilar immune-inflammatory-genetic substrate of each disease or by disease-specific drugs or by other accumulated comorbidities is not clarified by this study. In the analysis we took into consideration all major and most underlying classical CVD risk factors [29] but not other emerging CVD risk factors (such as prothrombotic markers, C-reactive protein). However, these are disease-related characteristics and are incorporated in the effect of each chronic disease. The present study comprised of Caucasian people and the results may not be extrapolated to other ethnicities. Finally, in the present study we used as comparator a population referred to the Cardiovascular Laboratory for established or suspected hypertension and not a community-based general population. For this reason the relative impact of each disease on subclinical atheromatosis when compared to the general population may be underestimated.

## Conclusions and Perspectives

This study evaluated both the femoral and carotid subclinical atheromatosis and showed that novel CVD risk factors such as RA disease and HIV infection, but also classical CVD risk factors have a differential impact on atheromatosis of the carotid and femoral artery. However, an equally high prevalence of isolated femoral subclinical atheromatosis in all conditions was found due to the accumulation of different comorbidities in each disease, whereas almost 50% of those with subclinical atheromatosis might be potentially reclassified at higher CVD risk due to the combined presence of femoral and carotid atheromatosis. In order to better estimate the global burden of arterial atherosclerosis [17], our findings promote the concept of mapping [59] at multiple easily accessible arterial sites, as the right-way to go forward towards the individualization and optimization of CVD risk management. Prospective CVD event-driven studies should further evaluate this concept.
